# Deep learning image reconstruction algorithm for carotid dual-energy computed tomography angiography: evaluation of image quality and diagnostic performance

**DOI:** 10.1186/s13244-022-01308-2

**Published:** 2022-11-26

**Authors:** Chenyu Jiang, Dan Jin, Zhuoheng Liu, Yan Zhang, Ming Ni, Huishu Yuan

**Affiliations:** 1 Department of Radiology, Peking University Third Hospital, Beijing, China; 2 CT Research Center, GE Healthcare China, 1 South Tongji Road, Beijing, China

**Keywords:** Dual-energy computed tomography, Deep learning, Image reconstruction

## Abstract

**Objectives:**

To evaluate image quality and diagnostic performance of carotid dual-energy computed tomography angiography (DECTA) using deep learning image reconstruction (DLIR) compared with images using adaptive statistical iterative reconstruction-Veo (ASIR-V).

**Methods:**

Carotid DECTA datasets of 28 consecutive patients were reconstructed at 50 keV using DLIR at low, medium, and high levels (DLIR-L, DLIR-M, and DLIR-H) and 80% ASIR-V algorithms. Mean attenuation, image noise, signal-to-noise ratio (SNR), and contrast-to-noise ratio (CNR) at different levels of arteries were measured and calculated. Image quality for noise and texture, depiction of arteries, and diagnostic performance toward carotid plaques were assessed subjectively by two radiologists. Quantitative and qualitative parameters were compared between the ASIR-V, DLIR-L, DLIR-M, and DLIR-H groups.

**Results:**

The image noise at aorta and common carotid artery, SNR, and CNR at all level arteries of DLIR-H images were significantly higher than those of ASIR-V images (*p* = 0.000–0.040). The quantitative analysis of DLIR-L and DLIR-M showed comparable denoise capability with ASIR-V. The overall image quality (*p* = 0.000) and image noise (*p* = 0.000–0.014) were significantly better in the DLIR-M and DLIR-H images. The image texture was improved by DLR at all level compared to ASIR-V images (*p* = 0.000–0.008). Depictions of head and neck arteries and diagnostic performance were comparable between four groups (*p* > 0.05).

**Conclusions:**

Compared with 80% ASIR-V, we recommend DLIR-H for clinical carotid DECTA reconstruction, which can significantly improve the image quality of carotid DECTA at 50 keV but maintain a desirable diagnostic performance and arterial depiction.

## Key points

- DLIR is suitable for the reconstruction of dual-energy computed tomography angiography.
- DLIR-H generated the best image quality regarding image noise and texture.
- DLIR images have similar arterial depictions and diagnostic performance to vascular lesions to ASIR-V.

## Introduction

Carotid atherosclerosis is the most prevalent chronic disease in the developed world [1, 2]. Computed tomography angiography (CTA) offers excellent efficiency to detect carotid and intracranial arterial pathology [3], which has become an established second-line technique for screening carotid artery disease [4]. According to a recent survey, between 2007 and 2017 in the emergency department, the growth of head and neck CTA far outraced the growth of other modalities with a compound annual growth rate of around 24% [5]. This rapid increase in CTA examination aroused concern about the risk of radiation exposure and iodine dose.

To address this issue, various iterative reconstruction (IR) algorithms are developed to obtain a better image quality with lower radiation dose, such as hybrid IR that blend IR with filtered back projection (FBP) [6–9]. Adaptive statistical iterative reconstruction-Veo (ASIR-V, GE Healthcare) is a vendor-specific hybrid IR allowing more aggressive image noise and radiation dose reduction with a much short reconstruction time than other IR techniques [10]. However, ASIR-V shares the limitation with other IR techniques, that is, it still hardly reached a trade-off between image noise and unnatural image texture [7–9]. Recently, a vendor-specific deep neural network–based recon engine (TrueFidelity™2.0; GE Healthcare) for image denoising algorithms, termed deep learning image reconstruction (DLIR), has been proposed for CT image reconstruction, which is trained with artifact-free FBP datasets of both phantoms and patients to differentiate noise from signals [11]. DLIR allows emulating standard-dose FBP image texture while providing low image noise, artifact suppression, and highly sensitive detectability with high resolution [12–16]. Its utility has been demonstrated in both single-energy CT and dual-energy CT scans until most recently [17–19]; however, its adaption to dual-energy CTA (DECTA) has not been evaluated yet.

The different predicted monoenergetic kiloelectron volt (keV) levels, also known as virtual monochromatic images (VMIs) is a common postprocessing option for DECTA. Lower energy VMIs enable the enhancement of the attenuation of iodine contrast of CTA images by shifting the energy of the photons toward the k-edge of iodine at 33 keV, at the expense of a dramatically increased noise [20, 21]. A recent carotid DECTA study using IR algorithm indicated VMIs at 50 keV energy level not only have a superior CNR than those of single-energy scans with 120 kV, but also is the optimal keV energy level for subjective assessment for carotid DECTA [22].

We hypothesized that with the application of DLIR algorithm in carotid DECTA, low energy VMI may possess more favorable image noise and texture while maintain better signal-to-noise ratio (SNR) and contrast-to-noise ratio (CNR) than ASIR-V. Therefore, this study aimed to evaluate the image quality and diagnostic performance toward head and neck arterial pathology of VMI at 50 keV from carotid DECTA using the DLIR algorithm, compare with those of images reconstructed with ASIR-V.

## Materials and methods

This study was approved by the institutional review board at our institution. Written informed consent was obtained from all patients, and retrospective analyses were performed using a prospective cohort.

### Participants

Forty-four consecutive patients who underwent carotid DECTA (Revolution CT; GE Healthcare that was able to reconstruct both ASIR-V and DLIR images) between November 2021 and December 2021 were included in the study. Among them 3 patients had metal implants after neck surgery; 5 patients had carotid stents; 5 patients had extensive thrombosis were excluded as accurate intravascular evaluations were not possible due to metal artifacts and thrombosis; and raw data of 3 patients were lost. Finally, 28 patients were included in this study.

### Dual-energy CT technique

All examinations were performed by a fast kilovoltage-switching DECT scanner (Revolution CT; GE Healthcare) with carotid DECTA imaging parameters: 80/140 kV peak tube voltage, 12 HU noise index at 5-mm section collimation, variable tube current (GSI Assist; GE Healthcare); detector configuration, 128 detectors with 0.625 mm section thickness; 80 mm beam collimation, 0.5 s rotation time, 0.984:1 pitch, 36 cm display field-of-view. For contrast enhancement, nonionic-iodinated contrast agent (370 mgI/mL, Omnipaque 350, GE Healthcare, Shanghai, China) was injected intravenously into the right cubital at a rate of 3.5 mL/s using an automatic injector with a bolus of 40 mL and followed by 30-mL saline flush at the same injection rate. CTA was triggered by a bolus-tracking program (trigger point: the ascending aorta, trigger threshold: 120 HU) with a 5-s delay in image acquisition. CT images were obtained from the cranial crest to the aortic arch in the craniocaudal direction.

### Imaging reconstruction

The raw data were reconstructed at 0.625 mm section thickness using 80% ASIR-V (IR 80% + FBP 20%) and DLIR algorithm at three selectable strength levels (low, DLIR-L; medium, DLIR-M; high, DLIR-H) graded by noise reduction capability. All reconstructions used standard kernel. All reconstructed images were processed into VMI at 50 keV using GSI Viewer software (Advantage Workstation, version 4.7, GE Healthcare).

### Quantitative image analysis

Image analysis and measurement were performed on GSI Viewer software by one radiologist who was blind to images reconstruction algorithms. ROIs were manually placed on the axial images at levels of aortic arch (AA), common carotid artery (CCA), internal carotid artery (ICA), and vertebral artery (VA) at the dominating side to measure the mean attenuation (HU) and noise (SD) values. ROIs should cover the central parts of the arteries as much as possible while avoiding arterial wall, plaques, and severe artifacts. To calculate the CNR of the target vessel, HU values of sternocleidomastoid were also measured at the level of hyoid. Values of SNR and CNR were calculated using the equation: SNR = target HU / target SD and CNR = (target HU − muscle HU)/target SD.

### Qualitative image analysis

In order to standardize qualitative analysis, two experienced radiologists were trained for image quality evaluation prior to qualitative image analysis. The VMIs dataset reconstructed by 80% ASIR-V, DLIR-L, DLIR-M, and DLIR-H were hanged in randomized order at GSI Viewer software to each radiologist without any annotations. All images were presented with preset window set: level, 100 HU and width, 800 HU, radiologists were allowed to adjust the window set during evaluations.

Two radiologists independently reviewed overall image quality for noise and texture of each image using a 5-point Likert scale: 5 = excellent for the best image quality, 4 = favorable (no influence on image interpretation), 3 = acceptable for diagnosis (possible influence); 2 = suboptimal (mild influence), and 1 = poor (impairing diagnosis). Two radiologists further rated the arterial depiction of head and neck artery using a 5-point Likert scale (Table 1) as published previously [23], according to vascular edge and subjective contrast to noise: 5 = very sharp edge with high contrast; 4 = sharp edge with satisfied contrast; 3 = minimal blurring edge with suboptimal contrast; 2 = blurring edge with markedly suboptimal subjective contrast; 1 = unacceptable blurring and contrast. In addition to CCA, ICA, VA and basilar artery (BA), we detailed the assessment of intracranial arteries, including anterior cerebral artery (ACA), middle cerebral artery (MCA), and posterior cerebral artery (PCA), at different segments according to Netter's cerebrovascular classification [24].

**Table 1:** Subjective image quality analysis

	ASIR	DLIR-L	DLIR-M	DLIR	*p* value	*κ* values
*Image noise*						
Reader 1	3.4 ± 0.5 (3–4)	3.3 ± 0.6 (2–4)	3.7 ± 0.4* (3–4)	4.5 ± 0.5* (4–5)	0.000	0.53
Reader 2	3.5 ± 0.5 (3–4)	3.5 ± 0.6 (2–4)	3.7 ± 0.4* (3–4)	4.4 ± 0.5* (4–5)	0.000	
*Image texture*						
Reader 1	3.5 ± 0.6 (2–4)	3.8 ± 0.5* (3–5)	3.8 ± 0.4* (3–5)	4.4 ± 0.6* (3–5)	0.000	0.57
Reader 2	3.5 ± 0.6 (2–4)	3.7 ± 0.4* (3–4)	3.8 ± 0.4* (3–4)	4.6 ± 0.6* (3–5)	0.000	
*Overall image quality*						
Reader 1	3.4 ± 0.4 (3–4)	3.6 ± 0.4 (3–5)	3.8 ± 0.3* (3–5)	4.4 ± 0.3* (3–5)	0.000	0.44
Reader 2	3.5 ± 0.3 (3–4)	3.6 ± 0.3 (3–4)	3.8 ± 0.3* (3–4)	4.4 ± 0.3* (4–5)	0.000	

*Mean statistical higher than ASIR-V

ASIR, adaptive statistical iterative reconstruction-Veo; DLIR, deep learning image reconstruction (low strength, DLIR-L; medium strength, DLIR-M; high strength, DLIR-H)

### Diagnostic evaluation

Moreover, two experienced radiologists who were blinded to the patient clinical data and images reconstruction algorithms were asked to evaluate the diagnostic performance to carotid plaques of the ASIR-V and DLIR images in consensus. Stenotic lesion was graded as the following criteria: 0 = no stenosis; 1 = mild (0–49%); 2 = moderate (50–69%); 3 = severe (70–99%); 4 = obstruction. According to composition variation, carotid plaques were divided into: 1, non-calcified plaques; 2, mixed plaque; 3, calcified plaques. There was an interval of 2 weeks between the evaluation of ASIR-V and DLIR images.

### Statistical analysis

All statistical analyses were performed on software SPSS version 26.0 (IBM). Image quantitative parameters including HU, SD, SNR and CNR were analyzed using repeated measures ANOVA with the Bonferroni post hoc test between the ASIR-V, DLIR-L, DLIR-M, and DLIR-H groups. For the qualitative analysis including, overall image quality, and subjective ratings of different arterial segments, the Friedman test was conducted to compare these subjective indicators among four groups. The paired Wilcoxon signed-rank test was performed for post hoc subgroup comparisons when a significant difference was found between the four groups. The McNemar test was used to compare the diagnostic performance of DLIR and ASIR-V images. Inter-observe agreement and the agreement of the diagnostic results between DLIR and ASIR-V images were tested using Cohen’s kappa test, using the following criteria: poor (*κ* < 0.4); moderate (*κ* = 0.41–0.60); good (*κ* = 0.61–0.80); excellent (*κ* = 0.81–1.00). A *p* value < 0.05 was considered to indicate statistical significance.

## Results

Twenty-eight patients comprising 16 males and 12 females were included in this study, with mean age 59.9 ± 12.6 years (range 33–81 years) and mean body mass index (BMI) 25.7 ± 3.1 kg/m^2^ (range 20.0–33.4 kg/m^2^).

### Quantitative image analysis

**Fig. 1:**
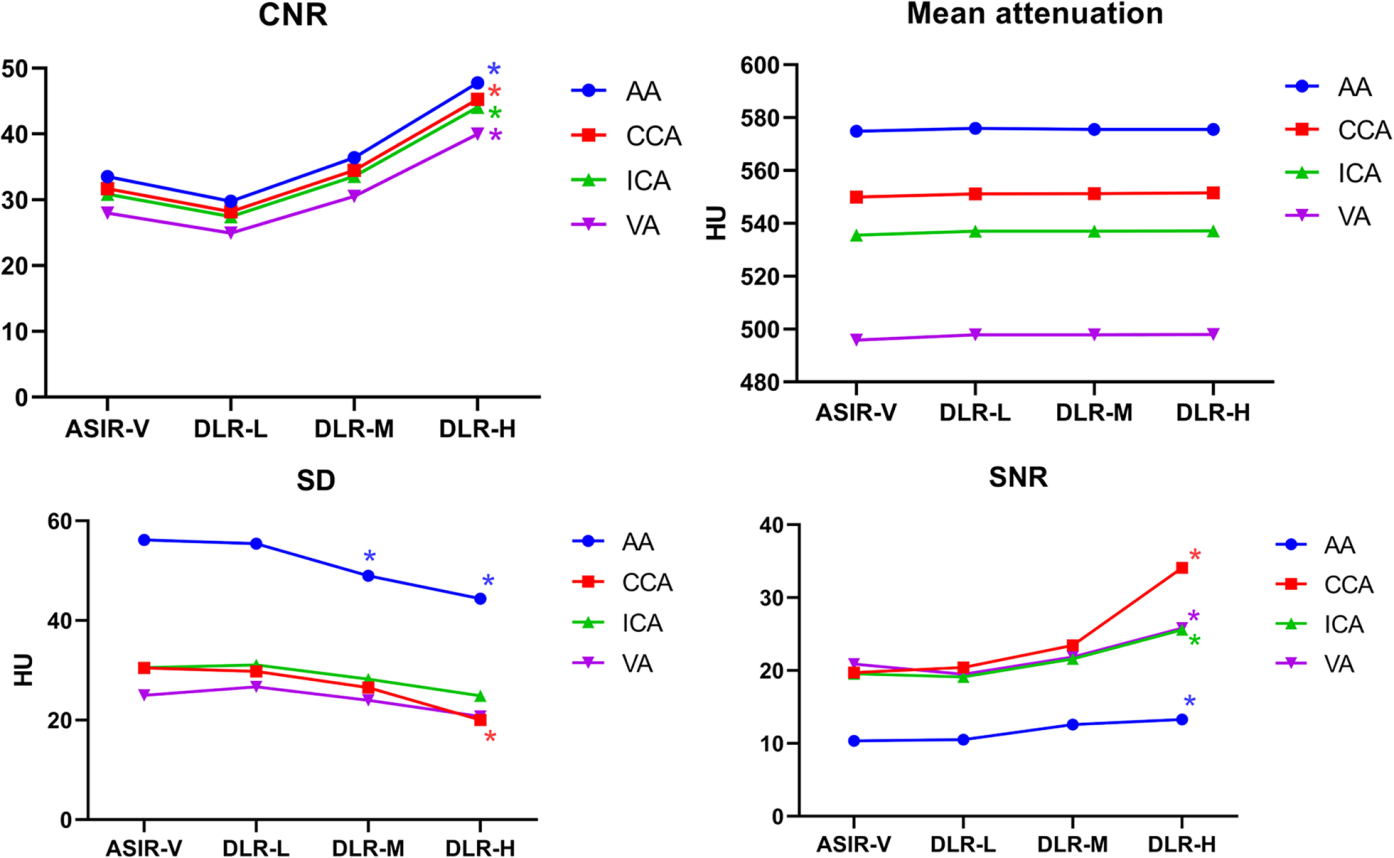
Quantitative image analysis for arteries at all level between four reconstructed images. *Mean statistical higher than ASIR-V

CT attenuation at levels of AA, CCA, ICA, and VA were comparable among the four groups. Difference of SNR, CNR at all arterial level, and SD at level of AA, CCA, and VA were noted between four reconstructed images (SNR: *p* = 0.000–0.015; CNR: all *p* = 0.000; SD: 0.001–0.027). However, the Bonferroni post hoc test shows SD only at level of AA and CCA in DLIR-H images lower than ASIR-V images (AA: 44.4 ± 7.9 vs 56.1 ± 7.9, *p* = 0.000 and CCA: 9.9 ± 10.4 vs 30.5 ± 10.8, *p* = 0.002), no difference was noted in SD of ICA and VA. More importantly, SNR (AA: 13.3 ± 3.1 vs 10.3 ± 1.9, *p* = 0.017; CCA: 34.1 ± 16.7 vs 19.7 ± 6.4, *p* = 0.000; ICA: 25.6 ± 10.6 vs 19.6 ± 6.8, *p* = 0.040; VA: 25.8 ± 7.5 vs 20.9 ± 5.8, *p* = 0.021) and CNR (47.8 ± 11.7 vs 33.5 ± 8.5, *p* = 0.000; 45.3 ± 11.1 vs 31.7 ± 8.2, *p* = 0.000; 44.1 ± 11.2 vs 30.8 ± 8.2, *p* = 0.000; 39.9 ± 9.1 vs 27.9 ± 6.9, *p* = 0.000) at all arterial level in DLIR-H significantly outmatched those of ASIR-V. Besides, the quantitative analysis of DLIR-L and DLIR-M showed comparable denoise capability with ASIR-V. (All quantitative analysis is shown in Fig. 1.)

### Qualitative image analysis

**Fig. 2:**
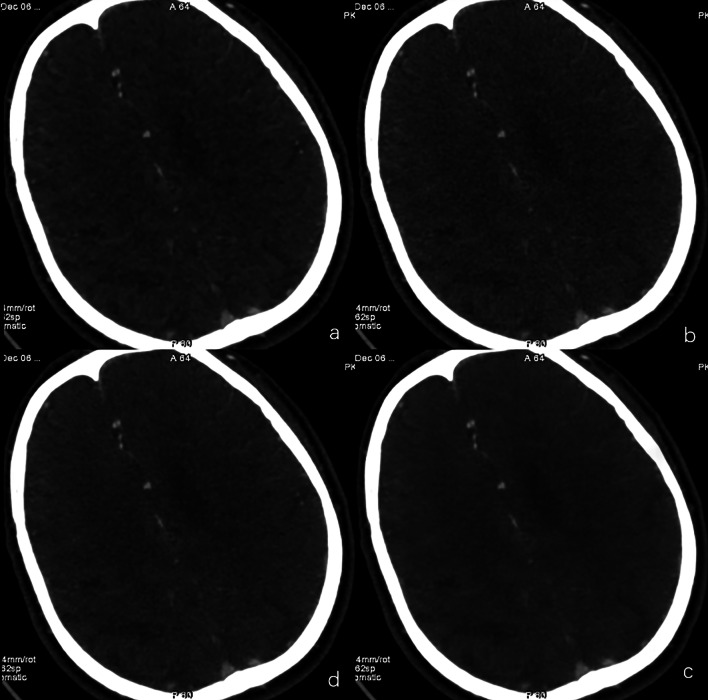
Qualitative image analysis for image noise and texture between four reconstructed images. A 71-year-old male with carotid artery occlusion. The 80% ASIR-V (**a**), DLIR-L (**b**), DLIR-M (**c**), and DLIR-H (**d**) are shown. The image noise of DLIR-L seems to be similar to that of ASIR-V, but the image of DLIR-M and DLIR-H is obviously better than ASIR-V. The over-smooth “plastic-looking” texture at centrum semiovale the most striking in the ASIR-V image, while the unnatural texture was reduced in all DLIR images, especially in DLIR-H

The results of image qualitative analysis are summarized in Table 1. Significant differences were noted in overall image quality, image noise, artifact, and noise texture among four groups (Fig. 2). In post hoc subgroup comparisons, compared with ASIR-V, images noise in DLIR-M and DLIR-H image was significantly lower (reader 1: *P*_DLIR-M_ = 0.002, *P*_DLIR-H_ = 0.000; reader 2: *P*_DLIR-M_ = 0.014, *P*_DLIR-H_ = 0.000), DLR at all strength levels were superior to ASIR-V in terms of image texture (reader 1: *P*_DLIR-L_ = 0.004 *P*_DLIR-M_ = 0.008, *P*_DLIR-H_ = 0.000; reader 2: *P*_DLIR-L_ = 0.005 *P*_DLIR-M_ = 0.004, *P*_DLIR-H_ = 0.000). The overall image quality of DLIR-M and DLIR-H is significantly better than that of ASIR-V (reader 1: *P*_DLIR-M_ = 0.000, *P*_DLIR-H_ = 0.000; reader 2: *P*_DLIR-M_ = 0.000, *P*_DLIR-H_ = 0.000).

**Fig. 3:**
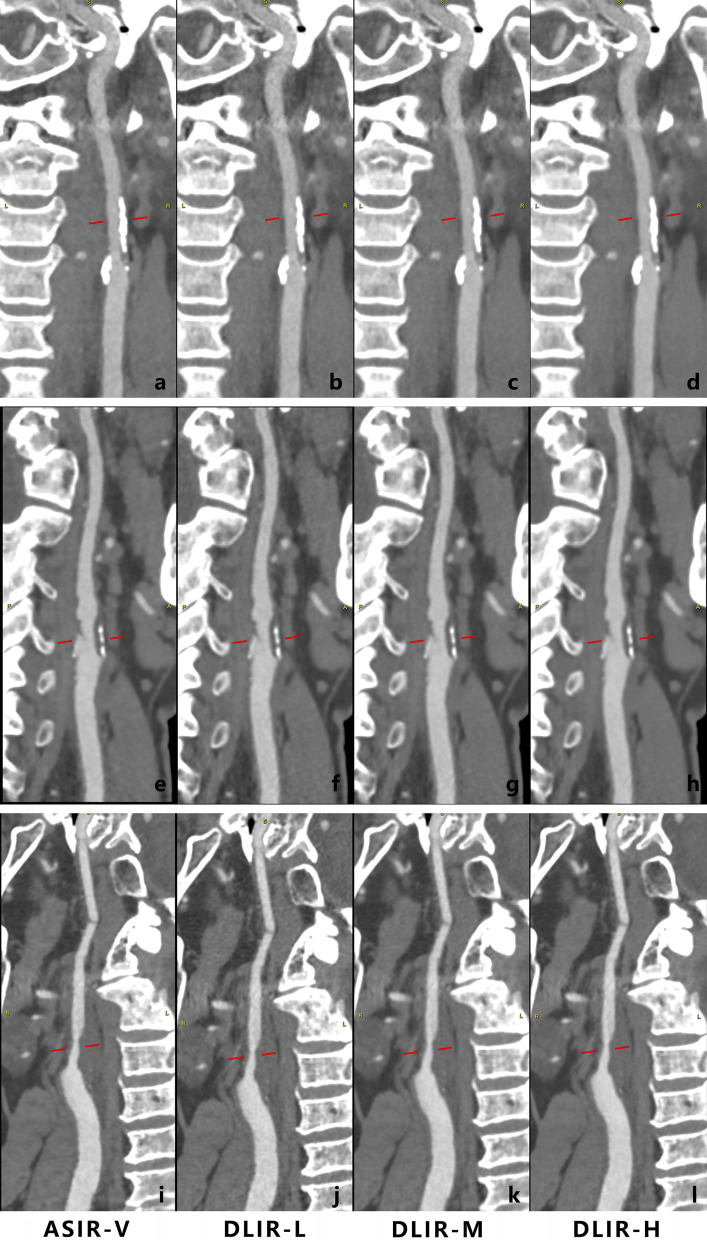
Visualization carotid plaques of four different reconstructed images. **a**–**d** Calcified plaque at proximal carotid; **e**–**h** mixed plaque at carotid bifurcation; **i**–**l** non-calcified plaque at carotid bifurcation. The visualization of plaque composition and the estimation of stenosis gradation were almost identical between 80% ASIR-V and DLIR at all strength level

In evaluation of arteries depiction, almost all the vascular segments of the head and neck artery showed similar depiction (Fig. 3) in the four reconstructed images for both readers, except for ACA-A3 segment (Table 2). One reader believed the depiction of A3 in DLIR-H images was statistically higher than that of ASIR-V (*p* = 0.014), while no difference was noted by reader 2, despite moderate agreement between two readers (*κ* = 0.60). In the subjective analysis of image quality and arterial depiction, moderate-to-excellent agreement was noted between the two readers (*κ* value ranged from 0.44 to 0.92).

**Table 2:** Arterial depiction on four reconstructed images

ASIR-V (Reader 1) (Reader 2)	DLIR-L (Reader 1) (Reader 2)	DLIR-M (Reader 1) (Reader 2)	DLIR-H (Reader 1) (Reader 2)	*p* value	*κ* value
*CCA*					
4.5 ± 0.6 (3–5)	4.4 ± 0.7 (2–5)	4.5 ± 0.6 (3–5)	4.6 ± 0.6 (3–5)	0.096	0.78
4.4 ± 0.6 (3–5)	4.4 ± 0.7 (2–5)	4.4 ± 0.6 (3–5)	4.5 ± 0.5 (3–5)	0.072	
*ICA*					
4.4 ± 0.6 (3–5)	4.4 ± 0.7 (3–5)	4.4 ± 0.6 (3–5)	4.4 ± 0.6 (3–5)	0.261	0.75
4.3 ± 0.7 (3–5)	4.3 ± 0.6 (3–5)	4.2 ± 0.6 (3–5)	4.3 ± 0.6 (3–5)	0.300	
*VA*					
4.2 ± 0.6 (3–5)	4.1 ± 0.7 (2–5)	4.2 ± 0.6 (3–5)	4.3 ± 0.6 (3–5)	0.080	0.67
4.2 ± 0.6 (3–5)	4.0 ± 0.7 (2–5)	4.1 ± 0.6 (3–5)	4.2 ± 0.5 (3–5)	0.181	
*BA*					
4.1 ± 0.5 (3–5)	4.0 ± 0.6 (3–5)	4.1 ± 0.5 (3–5)	4.1 ± 0.5 (3–5)	0.511	0.83
4.0 ± 0.6 (3–5)	4.0 ± 0.7 (3–5)	4.0 ± 0.6 (3–5)	4.1 ± 0.6 (3–5)	0.429	
*ACA*					
A1–A2					
4.5 ± 0.6 (3–5)	4.5 ± 0.6 (3–5)	4.5 ± 0.6 (3–5)	4.5 ± 0.6 (3–5)	0.779	0.85
4.4 ± 0.6 (3–5)	4.5 ± 0.6 (3–5)	4.5 ± 0.6 (3–5)	4.5 ± 0.6 (3–5)	0.724	
A3					
3.3 ± 0.8 (2–4)	3.3 ± 0.8 (2–4)	3.4 ± 0.7 (2–4)	3.5 ± 0.5* (3–4)	0.002	0.60
3.2 ± 0.6 (2–4)	3.3 ± 0.6 (2–4)	3.3 ± 0.6 (2–4)	3.3 ± 0.5 (2–4)	0.753	
A4					
2.2 ± 0.7 (1–4)	2.2 ± 0.7 (1–4)	2.3 ± 0.8 (1–4)	2.3 ± 0.9 (1–4)	0.112	0.89
2.1 ± 0.7 (1–3)	2.1 ± 0.7 (1–3)	2.2 ± 0.8 (1–3)	2.3 ± 0.7 (1–3)	0.248	
*MCA*					
M1–M2					
4.3 ± 0.6 (3–5)	4.3 ± 0.6 (3–5)	4.3 ± 0.6 (3–5)	4.2 ± 0.6 (3–5)	0.896	0.78
4.3 ± 0.6 (3–5)	4.3 ± 0.6 (3–5)	4.3 ± 0.6 (3–5)	4.3 ± 0.6 (3–5)	0.494	
M3					
3.6 ± 0.6 (2–5)	3.6 ± 0.7 (2–5)	3.6 ± 0.6 (2–5)	3.6 ± 0.6 (2–5)	0.261	0.84
3.5 ± 0.6 (2–4)	3.4 ± 0.6 (2–4)	3.5 ± 0.6 (2–4)	3.5 ± 0.6 (2–5)	0.284	
M4					
3.2 ± 0.7 (2–4)	3.1 ± 0.8 (1–4)	3.2 ± 0.7 (1–4)	3.2 ± 0.8 (1–4)	0.392	0.92
3.2 ± 0.7 (2–4)	3.1 ± 0.9 (1–4)	3.2 ± 0.8 (1–4)	3.1 ± 0.8 (1–4)	0.644	
*PCA*					
P1–P2					
4.2 ± 0.6 (3–5)	4.2 ± 0.6 (3–5)	4.2 ± 0.6 (3–5)	4.2 ± 0.6 (3–5)	1.000	0.84
4.3 ± 0.6 (3–5)	4.3 ± 0.6 (3–5)	4.3 ± 0.6 (3–5)	4.3 ± 0.6 (3–5)	0.392	
P3					
3.1 ± 0.7 (1–4)	3.1 ± 0.7 (1–4)	3.1 ± 0.7 (1–4)	3.2 ± 0.7 (2–4)	0.332	0.86
3.0 ± 0.6 (1–4)	3.0 ± 0.7 (1–4)	3.0 ± 0.6 (1–4)	3.0 ± 0.7 (1–4)	0.675	
P4					
2.0 ± 0.7 (1–4)	2.0 ± 0.7 (1–4)	2.0 ± 0.7 (1–4)	2.2 ± 0.7 (1–4)	0.068	0.82
2.1 ± 0.8 (1–4)	2.0 ± 0.7 (1–4)	2.1 ± 0.8 (1–4)	2.1 ± 0.6 (1–3)	0.463	

*Mean statistical higher than ASIR-V

AA, aortic arch; CCA, common carotid artery; ICA, internal carotid artery; VA, vertebral artery; BA, basilar artery; ACA, anterior cerebral artery; MCA, middle cerebral artery; PCA, posterior cerebral artery. ASIR, adaptive statistical iterative reconstruction-Veo; DLIR, deep learning image reconstruction (low strength, DLIR-L; medium strength, DLIR-M; high strength, DLIR-H)

### Diagnostic evaluation

We found 35 stenotic lesions with various carotid plaque morphology and composition on each ASIR-V and DLIR image. As shown in Tables 3 and 4, DLIR of all strength and ASIR-V revealed no difference in determination of carotid plaque composition and gradation of stenosis with almost perfect diagnosis agreement (Table 3 for plaque composition, DLIR-L: 91.1%, for DLIR-M: 91.0%, for DLIR-H: 86.7%; Table 4 for stenosis gradation, DLIR-L: 68.0%, for DLIR-M: 80.0%, for DLIR-H: 80.3%).

**Table 3:** Determination of carotid plaque composition between ASIR-V and DLIR images

ASIR-V	DLIR-L (*n*)			DLIR-M (*n*)			DLIR-H (*n*)		
	Non-calcified	Mixed	Calcified	Non-calcified	Mixed	Calcified	Non-calcified	Mixed	Calcified
Non-calcified	14	2	0	15	1	0	15	1	0
Mixed	0	6	0	0	6	0	0	6	0
Calcified	0	0	13	0	1	12	0	2	11
*p* value	0.157			0.368			0.223		
*κ*-values	0.911			0.910			0.867		

ASIR, adaptive statistical iterative reconstruction-Veo; DLIR, deep learning image reconstruction (low strength, DLIR-L; medium strength, DLIR-M; high strength, DLIR-H)

**Table 4:** Estimation of stenosis gradation between ASIR-V and DLIR images

ASIR-V	DLIR-L				DLIR-M				DLIR-H			
	Mild	Moderate	Severe	Obstruction	Mild	Moderate	Severe	Obstruction	Mild	Moderate	Severe	Obstruction
Mild	6	0	0	0	5	1	0	0	5	1	0	0
Moderate	1	5	2	0	1	6	1	0	0	8	0	0
Severe	0	3	11	0	0	1	13	0	0	3	11	0
Obstruction	0	0	2	5	0	0	1	6	0	0	1	6
*p* value	0.362				0.801				0.172			
*κ*-values	0.680				0.800				0.803			

ASIR, adaptive statistical iterative reconstruction-Veo; DLIR, deep learning image reconstruction (low strength, DLIR-L; medium strength, DLIR-M; high strength, DLIR-H)

## Discussion

Recently, aortic DECTA with a reduced iodine dose protocol had demonstrated clinical utility of DLIR algorithm in DECTA [19]. In this study, we further evaluated the utility of DLIR algorithm in head and neck DECTA at 50 keV by image objective parameters and subjective evaluation including general image quality in terms of image noise, image texture, depiction of arteries at different levels, and diagnostic performance with conventional ASIR-V. Our results indicated that DLIR-H algorithm could reduce carotid DECTA image noise and improving image quality while maintaining similar arterial depiction and diagnostic performance, compared to 80% ASIR-V.

ASIR-V as a vendor-specific hybrid IR has a major limitation of the unnatural textures [9], which was a typical finding in IR algorithms and was mentioned as “blotchy” “plastic-looking” [6, 7, 9]. Moreover, in past experience with CT reconstruction using ASIR-V with a blending factor of 30–60% the image blur due to noise texture manifested with the blending factor increases in spite of enhanced noise reduction [13, 14, 25, 26]. We adopted using high blending factors of 80% ASIR-V as reference standard to test the capability of DLIR algorithm for two reasons. Firstly, 80% ASIR-V is the routine image reconstruction algorithm for GE Healthcare CT system at our organization. Secondly, ASIR-V with blending factor of 40% was used for comparison in all former DLIR algorithms of DECT study [18, 19] and the comparison of image texture was also omitted, which makes further comparison between ASIR-V with high blending factor and DLIR algorithm necessary.

In subjective evaluation, overall image quality and image noise of DLIR-M and DLIR-H were preferable to 80% ASIR-V which was consistent with the previous study. Moreover, all strength of DLIR had finer image texture compared to 80% ASIR-V. Although the noise texture of images is usually evaluated using noise-power spectrum (NPS), here, we only perform subjective evaluation, because NPS was usually only used in phantom studies [27, 28], and we consider it more clinically significant to evaluate the influence of such unnatural texture on image interpretation. The subjective evaluation of image texture in this study suggested that DLIR might show similar image texture patterns to FBP with more clinical applicability, compared to high-level ASIR-V.

In our quantitative analysis, DLIR-H algorithm significantly improved arteries SNR and CNR at all segments as expected. However, the image noise value of ICA and VA seemed to decrease significantly compared with 80% ASIR-V, which was inconsistent with previous studies [13, 19]. The small changes in ROI placement can significantly alter the measurement results at the segment of ICA and VA with a small diameter may be a reasonable explanation. Although DLIR-M had some advantages in subjective image evaluation, the quantitative parameters of DLIR-M were comparable to that of 80% ASIR-V. As for DLIR-L, no advantage over 80% ASIR-V images was noted in both qualitative and quantitative analyses.

Jansen et al., as well as subsequent studies [12, 14, 17, 19], warned the possibility of DLIR-H obscuring small lesions and suboptimal detail evaluation of small vessels compared with ASIR-V. But our results seemed to contradict this view; the arterial depictions at almost all arterial segment were comparable between four reconstruction algorithms, even a reader thought that depiction of ACA-A3 segment in DLIR-H image was the most superior. We believed this was benefit of image noise reduction, generally outweighed blurring of small lesion or vessel, which also was mentioned by Jansen et al. [12]. Besides, it was worth noting that the factor of ASIR-V used by the previous study was relatively low (40–60%), so noise texture was no as significantly as 80% ASIR-V to influence observation of small arteries in our study. Hence, it is reasonable to believe that improvement in image texture and noise reduction by DLIR-H compensated for the blurring effect on intracranial small arteries.

**Fig. 4:**
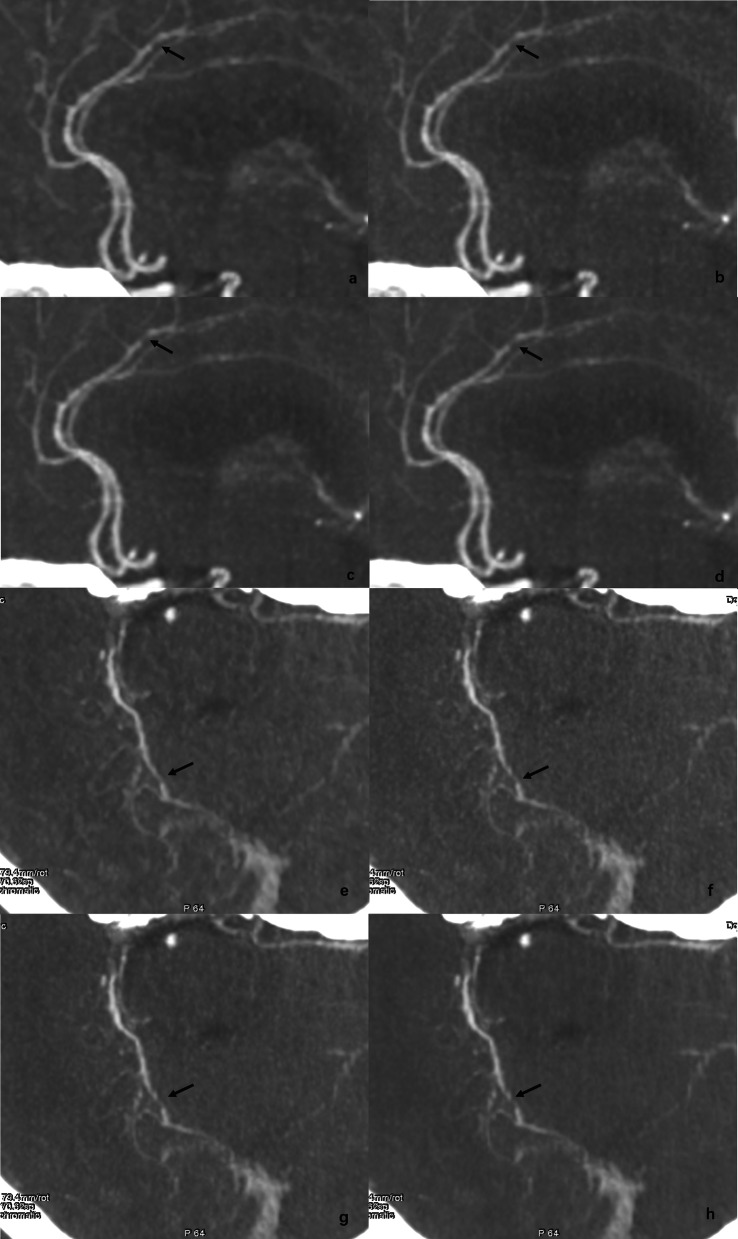
Examples of overestimated intracranial arterial stenosis on DLIR images. ASIR-V (**a**, **e**), DLIR-L (**b**, **f**), DLIR-M (**c**, **g**), DLIR-H (**d–h**) of a 69-year-old woman (**a**–**d**) and a 71-year-old male (**e**–**h**) with cerebral atherosclerosis are shown. 80% ASIR-V and DLIR images show similar depiction of the proximal and distal cerebral arteries. Moderate-to-severe focal stenosis at segment A3 of right anterior cerebral artery and segment P3 of right posterior cerebral artery are noticed (black arrow). The residual lumens at the stenosis are clearly visible in the ASIR-V images, while in the high-level DLIR image, residual lumens are vaguely visible

Our study had another strength; we evaluated the diagnostic performance of DECTA based on DLIR algorithm for variation of carotid plaque. Although we could not evaluate the diagnostic accuracy of images based on different algorithms due to the lack of a gold standard for composition of carotid plaque and stenosis gradation, the nearly uniform diagnostic performance between DLIR and ASIR-V images suggested that DLIR images are sufficient for visualization of actual carotid plaques under clinical conditions. Meanwhile, we also noticed an interesting phenomenon: DLIR-H image tends to overestimate the stenosis degree of small vessels (Fig. 4). This may be due to the blurring effect of the high level of DLIR on the residual lumen at the stenosis [12]. This phenomenon was only observed in certain cases of severe intracranial arterial stenosis, so it did not affect the detection of positive vascular lesions. Further study is desired to evaluate this subtle effect of high-level DLIR on characterization of small vessel stenosis.

This study had several limitations. First, the sample size of this study was relatively small. Second, further studies of task-specific lesion detection are necessary to determine the diagnostic accuracy of DLIR. Three, apart from VMI, other postprocessing techniques of DECT, such as iodine quantification, effective atomic number map, and material decomposition, were not involved in this study. The reliability of these postprocessing techniques in DLIR-based DECT images still needs further verification. Last, our CT system and algorithm were vendor specific. Algorithms and results may vary among vendors.

## Conclusion

DLIR is promising for DECTA reconstruction which can significantly reduce image noise, improve the image quality of carotid DECTA at 50 keV, but maintain a desirable diagnostic performance. Above all, compared with 80% ASIR-V algorithm, we recommend DLIR-H for carotid DECTA reconstruction. DLIR-M is an acceptable option.

## Data Availability

The datasets used and/or analyzed during the current study are available from the corresponding author on reasonable request.

## Abbreviations

AA: Aortic arch
ASIR-V: Adaptive statistical iterative reconstruction-Veo
CCA: Common carotid artery
CNR: Contrast-to-noise ratio
CTA: Computed tomography angiography
DECTA: Dual-energy computed tomography angiography
DLIR: Deep learning image reconstruction
DLIR-L, DLIR-M, DLIR-H: DLIR at low, medium, and high levels
FBP: Filtered back-projection
ICA: Internal carotid artery
IR: Iterative reconstruction
keV: Kiloelectron volt
NPS: Noise power spectrum
SNR: Signal-to-noise ratio
VA: Vertebral artery
VMI: Virtual monochromatic images
